# Cortical networks show characteristic recruitment patterns after somatosensory stimulation by pneumatically evoked repetitive hand movements in newborn infants

**DOI:** 10.1093/cercor/bhac373

**Published:** 2022-11-11

**Authors:** Eero Ahtola, Susanna Leikos, Anna Tuiskula, Leena Haataja, Eero Smeds, Harri Piitulainen, Veikko Jousmäki, Anton Tokariev, Sampsa Vanhatalo

**Affiliations:** Department of Clinical Neurophysiology, BABA Center, Pediatric Research Center, Children’s Hospital and HUS Diagnostics, Helsinki University Hospital and University of Helsinki, Helsinki, 00029 HUS, Finland; Department of Neuroscience and Biomedical Engineering, Aalto University School of Science, Espoo, 00076 AALTO, Finland; Department of Clinical Neurophysiology, BABA Center, Pediatric Research Center, Children’s Hospital and HUS Diagnostics, Helsinki University Hospital and University of Helsinki, Helsinki, 00029 HUS, Finland; Department of Clinical Neurophysiology, BABA Center, Pediatric Research Center, Children’s Hospital and HUS Diagnostics, Helsinki University Hospital and University of Helsinki, Helsinki, 00029 HUS, Finland; Department of Pediatric Neurology, Children’s Hospital, Helsinki University Hospital and University of Helsinki, Helsinki, 00029 HUS, Finland; Department of Pediatric Neurology, Children’s Hospital, Helsinki University Hospital and University of Helsinki, Helsinki, 00029 HUS, Finland; Children’s Hospital and Pediatric Research Center, Helsinki University Hospital and University of Helsinki, Helsinki, 00029 HUS, Finland; Department of Neuroscience and Biomedical Engineering, Aalto University School of Science, Espoo, 00076 AALTO, Finland; Faculty of Sport and Health Sciences, University of Jyväskylä, Jyväskylä, 40014, Finland; Aalto NeuroImaging, Department of Neuroscience and Biomedical Engineering, Aalto University, Espoo, 00076 AALTO, Finland; Department of Clinical Neurophysiology, BABA Center, Pediatric Research Center, Children’s Hospital and HUS Diagnostics, Helsinki University Hospital and University of Helsinki, Helsinki, 00029 HUS, Finland; Department of Clinical Neurophysiology, BABA Center, Pediatric Research Center, Children’s Hospital and HUS Diagnostics, Helsinki University Hospital and University of Helsinki, Helsinki, 00029 HUS, Finland; Department of Physiology, University of Helsinki, Helsinki, 00014, Finland

**Keywords:** corticokinematic coherence, EEG, functional networks, passive movement stimulation, perinatal asphyxia

## Abstract

Controlled assessment of functional cortical networks is an unmet need in the clinical research of noncooperative subjects, such as infants. We developed an automated, pneumatic stimulation method to actuate naturalistic movements of an infant’s hand, as well as an analysis pipeline for assessing the elicited electroencephalography (EEG) responses and related cortical networks. Twenty newborn infants with perinatal asphyxia were recruited, including 7 with mild-to-moderate hypoxic–ischemic encephalopathy (HIE). Statistically significant corticokinematic coherence (CKC) was observed between repetitive hand movements and EEG in all infants, peaking near the contralateral sensorimotor cortex. CKC was robust to common sources of recording artifacts and to changes in vigilance state. A wide recruitment of cortical networks was observed with directed phase transfer entropy, also including areas ipsilateral to the stimulation. The extent of such recruited cortical networks was quantified using a novel metric, Spreading Index, which showed a decrease in 4 (57%) of the infants with HIE. CKC measurement is noninvasive and easy to perform, even in noncooperative subjects. The stimulation and analysis pipeline can be fully automated, including the statistical evaluation of the cortical responses. Therefore, the CKC paradigm holds great promise as a scientific and clinical tool for controlled assessment of functional cortical networks.

## Introduction

A pertinent evaluation of the newborn infant’s cortical function is a persisting challenge for both neurodevelopmental science and pediatric clinical work. An essential aspect of this challenge is the lack of ability to cooperate combined with limited behavioral repertoire, especially in the infants with acute medical adversities. Yet, it has been clearly established that estimates of the early brain function, such as electrophysiological measures of the cortical function via electroencephalography (EEG), would provide the most sensitive prediction of neurological recovery or later neurobehavioral development (Walsh et al. 2011; Awal et al. 2016; Fogtmann et al. 2017). Currently, early electrophysiological assessment after medical adversities is focusing on the recovery of normal EEG background patterns or the emergence of the sleep–wake cycle (Walsh et al. 2011; Olischar et al. 2013; El-Dib et al. 2014), both of which are considered to be surrogate markers of the global brain health. In addition, a delayed or absent somatosensory response in the primary somatosensory cortex to electrical stimulation of a peripheral nerve is known to predict clinical outcome accurately in severe disabilities (Nevalainen et al. 2017; Pittet-Metrailler et al. 2020).

General interest in cortical electrophysiology is increasingly focused on the higher-level brain functions, such as attention, visuospatial skills, language, and memory. It is well established (e.g. Bassett and Bullmore 2009; Jung et al. 2018) that the higher brain functions are essentially network processes, and they rely on large-scale cortico-cortical interactions which can be measured using electrophysiological modalities, such as EEG or magnetoencephalography (MEG). Such functional networks can also be observed in sleeping infants, and recent studies have shown many clinically meaningful changes in the cortico-cortical network properties, e.g. frequency-specific patterns of dysconnectivity, after preterm birth (Tokariev et al. 2019; Yrjölä et al. 2022) or fetal drug exposures (Tokariev et al. 2022) for instance. There is, however, a striking mismatch between the currently available clinical methodology for newborn neurological assessment versus the need for controlled assessment of cortical networks in newborn infants.

Indirect evidence from long-latency somatosensory responses suggested that cortical information spread may correlate with later neurobehavioral outcomes (Rahkonen et al. 2013; Nevalainen et al. 2015); hence, the somatosensory system could potentially be utilized to assess cortico-cortical network integrity for predicting later neurodevelopment. This avenue is essentially blocked by practicalities: the conventional somatosensory response paradigm with electric nerve stimulation is unnatural and its combination with EEG recording comes with certain technical challenges, including potential issues with electrical safety. Meanwhile, many pragmatic issues compromise the response analysis by digital offline averaging as well as its essentially visual interpretation from the conventional, very small-amplitude somatosensory response waveforms.

Most of these challenges could be overcome by assessing sensory brain responses to well-controlled, naturalistic, repeated stimulation in frequency domain (Smeds et al. 2017; Ahtola et al. 2020), which yields responses robust to environmental noise (Bourguignon et al. 2016) and even allows statistical testing of the response significance (Kabdebon et al. 2022). We showed recently on an infant visual system study (Ahtola et al. 2020) that repeated stimulation can be used to measure the spread of the cortical responses from the primary visual cortex to other cortical areas. We now suggest that a comparable paradigm could also be viable with somatosensory stimuli for measurement of corticokinematic coherence (CKC) that emerges from the coupling between cortical activity and limb kinematics and reflects processing of the proprioceptor afference in the primary sensorimotor cortex (Piitulainen et al. 2013; Bourguignon et al. 2015). CKC methodology has already been widely developed for adults (Piitulainen et al. 2013, 2020; Bourguignon et al. 2015) and a recent proof-of-concept work using manual hand movement stimulation suggested its applicability in infants as well (Smeds et al. 2017).

Here, we set out to study how infants’ cortico-cortical networks can be examined in EEG responses to somatosensory stimuli using repeated naturalistic hand movements. We developed a clinically applicable, mechanical, pneumatic stimulation system for moving infant’s fingers accurately and automatically (cf. Piitulainen et al. 2015) and used the CKC paradigm (Bourguignon et al. 2011) to measure coupling between somatosensory afference and cortical activity (Smeds et al. 2017). In addition, cortico-cortical network recruitment related to CKC was studied by analyzing directional brain interactions using phase transfer entropy (PTE; Lobier et al. 2014) measures. Finally, we examined the technical robustness of the EEG responses to common sources of recording noise present in infant study environments, as well as the physiological sensitivity to infants’ vigilance state and clinical condition.

## Materials and methods

### Participants

We studied a cohort of 20 infants (10 females) that were recruited for another study exploring clinical signs of mild perinatal asphyxia (Tuiskula et al. 2022) after larger recruitment from the neonatal units of Helsinki University Children’s Hospital and Jorvi Hospital (Espoo, Finland) during 2018–2019. The infants were born full term at gestational age of 40.4 ± 1.2 weeks (mean ± standard deviation, SD), while their postnatal age at the time of the recording was 17.1 ± 11.3 days. The project was approved by the Ethics Committee of the Helsinki University Central Hospital, and parents or guardians of the participants gave an informed consent for the study and the publication.

The included infants met at least one of the following conditions: umbilical arterial cord pH below 7.10, 1-min Apgar score not exceeding 6, need for assisted ventilation or cardiopulmonary resuscitation at birth), but no other apparent reason for distress at birth. The exclusion criteria were the presence of known congenital anomaly or chromosomal abnormality, indication of another neurological condition, or obvious infection.

The infants were categorized into subgroups based on the evolution of neurological symptoms during their first 6 h after birth. The attending neonatologist diagnosed the presence and grade of hypoxic–ischemic encephalopathy (HIE1–3; Sarnat and Sarnat 1976). If the criteria of HIE was not fulfilled, the infant was categorized as HIE0 (perinatal asphyxia without hypoxic–ischaemic encephalopathy). The majority (*N* = 13) of the infants within the cohort belonged to the HIE0 category. Yet, 7 cases were diagnosed with either mild (HIE1; *N* = 5) or moderate (HIE2; *N* = 2) HIE. Infants with HIE3 were excluded from the study. This background information is presented individually in Supplementary Fig. S1 together with individual CKC measurement graphs.

The infants were also examined with magnetic resonance imaging (MRI) soon after the birth, at the median age of 2 weeks (interquartile range: 10–16 days), using a 3T scanner (Siemens Skyra, Siemens Healthcare, Erlangen, Germany). Our MRI protocol included axial T1- and T2-weighted images of the brain (details described in Tuiskula et al. 2022). A pediatric neuroradiologist, masked to the clinical condition, reviewed the MRIs following the scoring protocol of Weeke et al. (2018). The cohort was further categorized into subgroups based on whether they had abnormal findings in gray matter (GM; *N* = 4) or white matter (WM; *N* = 8). In total, 50% of the infants within the cohort (*N* = 10) were found to have abnormal findings in white and/or gray matter.

### E‌EG recording and movement stimulation

**Fig. 1:**
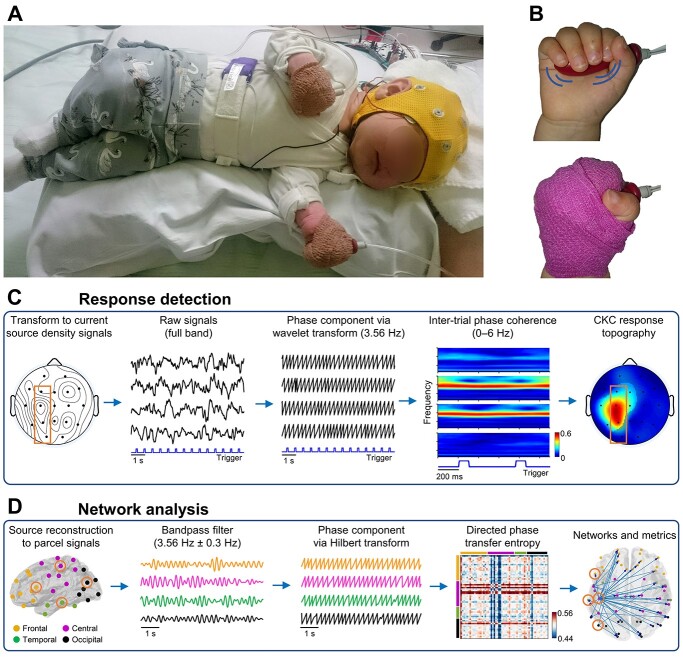
Experimental setup and illustrated analysis pipelines described in the method. A) The setup consisted of EEG recording using an EEG cap and balloons wrapped into the infant’s palms for delivering repetitive stimulation at 1.78 Hz frequency. B) The stimulation balloon before and after wrapping it inside the infant’s palm. The tube on the right side of the balloon was connected to the pump that was at about 2 m distance from the infant. C) Responses of CKC were detected from EEG using ITC at the first harmonic of the stimulator frequency. The EEG was made reference-free using current source density transformation, divided into epochs based on the trigger from the stimulator, while wavelet decomposition was used to extract the analytic phases of the signals at different frequencies. D) Cortical CKC networks were evaluated from the EEG transformed into cortical parcel signals and filtered around the response frequency (3.56 Hz). Directed PTE was calculated between analytic phases of all signal pairs and used to quantify directional connectivity in the functional networks between the parcels. The locations of the selected channels in the trace examples in (C) and (D) are marked with overlaid boundaries in the topographical figures.

The recording setup (Fig. 1A and B) consisted of the commercial EEG system and a custom-made movement actuator (“stimulator”). The stimulator included a pneumatic pump (FIA AB, Lund, Sweden; Supplementary Fig. S2) that could be programmed to generate periodic inflate/deflate cycles of a rubber bulb (“balloon”; taken from a regular laboratory pipette) at constant 1.78 Hz frequency (interstimulus interval 561 ± 2.9 ms as averages of mean and SD in the recordings). As the bulb was wrapped inside the infant’s palm, it exerted passive finger extensions and flexions (see Supplementary Video S1). The stimulator pump was stored in an aluminum case with layers of polyurethane foam to provide acoustic insulation so that it would attenuate most of the operating noise that could elicit a concomitant auditory response component.

The first harmonic of the fundamental stimulator frequency (3.56 Hz; later referred to as the “response frequency”) was used in the CKC analyses, because each movement cycle (comprising one inflation and one deflation of the pump) induces 2 somatosensory afference volleys arising primarily from the muscle afferents activated by extension and flexion of the fingers separately, and thus both likely contribute to the evoked brain response (Piitulainen et al. 2013, 2020). A total of 4 min of continuous movement stimulation was delivered to each hand, one at a time. Additionally, we recorded a control condition with the stimulator on, but the balloon removed from the infant’s hand to test whether any remaining pump noise (acoustic or electromagnetic) could lead to detectable brain responses when using the present kind of EEG analysis pipeline. These epochs also served as a general baseline in the statistical analyses when needed. For one infant, we could not perform this control recording at all due to technical challenges.

The EEG signal was acquired at 2 kHz with a clinical NicoletOne EEG system (Natus Medical Inc, Pleasanton, CA, USA) following the hospital’s routine protocol for neonatal EEG (as in Nevalainen et al. 2017, 2019). We used an EEG cap (Waveguard, ANT-Neuro, Berlin, Germany) with 21 sintered Ag/AgCl electrodes positioned consistently according to the international 10–20 standard: Fp1, Fp2, F7, F3, Fz (as a reference), F4, F8, M1, T3, C3, Cz, C4, T4, M2, T5, P3, Pz, P4, T6, O1, and O2. An analogue trigger output from the pump was connected to an auxiliary DC input of the EEG amplifier to provide timestamps for each inflation event, i.e. the synchronization between the EEG recording and the stimulation (see Fig. 1C and Supplementary Fig. S2). The data were exported to EDF+ files for further signal processing using customized scripts in MATLAB (MathWorks, Natick, MA, USA) environment.

### E‌EG preprocessing and visual inspection

The EEG was pre-filtered within 0.5 and 30 Hz with a combination of high-pass and low-pass digital filters (Butterworth IIR zero-phase forward and reverse filter, order 5), then resampled to 250 Hz to reduce the computational load, and finally segmented into 1,100 ms long epochs, always starting 200 ms before a stimulation trigger event (inflation) and ending 900 ms after it. Each segment was detrended by subtracting the local mean value from the signal. Alternative epoch durations were also tested during the development of the CKC algorithm. Results of this optimization are presented in Supplementary Fig. S3.

With average stimulation frequency of 1.78 Hz an epoch of the chosen length comprised 2 full stimulation cycles. No overlap between the consecutive epochs was allowed. The recordings were visually screened for high-amplitude artifacts by an experienced EEG reviewer (author SL). Only recognizable EEG was taken into further analysis as the epochs that coincided with the artifact segments were discarded (8.7% ± 11.9% of all epochs).

We wanted to assess how the prevailing vigilance states could affect the CKC, and hence, the sleep cycles within the recordings were visually scored based on EEG activity and trace alternant sleep characteristic. The recordings of right and left hand stimulations were classified independently using the standard sleep classes of newborns: active sleep (AS), quiet sleep (QS), and awake (Walsh et al. 2011). If none of the sleep classes covered more than 80% of the recoding alone, it was labeled as “undetermined” and excluded from the subsequent sleep state comparisons (25% of cases).

The overview of the EEG analysis workflow is presented in Fig. 1C and D. After general preprocessing steps, it is divided into 2 analytical branches: i) to determine whether (or not) the inspected recording contains a statistically significant CKC response (Fig. 1C) and ii) to evaluate the functional connectivity and cortical networks activated by the movement stimulation (Fig. 1D).

### Response detection from scalp signals using a CKC metric

The EEG responses evoked by the movement stimulation were estimated using CKC at the frequency band corresponding to the stimulation (both movement and control stimuli). Our implementation of CKC was based on inter-trial phase coherence (ITC) that yields a measure of phase-locked synchronization between repeated trials (i.e. epochs) in relation to the stimulus events (Tallon-Baudry et al. 1996; Delorme and Makeig 2004) using an equation:

$$\begin{equation*} \text{ITC}=\big|\frac{1}{N}\sum_{n=1}^N{e}^{i{\theta}_n}\big|, \end{equation*}$$

where θ*_n_* is instantaneous phase within a trial *n* and *N* is the total number of the trials. This approach deviates slightly from the conventional CKC paradigm, where the coherence is computed directly from the coupling between the movement kinematics (accelerometer) and cortical activity (M/EEG). Instead, we estimated the coherence with methods routinely used in evaluation of steady-state evoked potentials and event-related potentials in time–frequency plane (e.g. Brickwedde et al. 2020).

First, EEG was transformed to signals of current source density (CSD) using an algorithm based on spherical spline interpolation and scalp surface Laplacian (Perrin et al. 1989). CSD estimates are reference-free, and hence, the technique improves the accuracy of localization of the responsive areas at scalp level. To emphasize this, the transformation was performed with medium spline flexibility (*m* = 3) without smoothing (λ = 0).

We chose an analysis pipeline that would allow for adapting to varying temporal properties of the evoked oscillations (e.g. stationarity; Keil et al. 2022) To this end, we decided to calculate the ITC from the instantaneous phase components of the signal that we extracted using convolution with a set of Morlet wavelets that decompose the EEG into complex-valued time–frequency (*t*–*f*) plane. The wavelet frequency bands covered a range from 0.5 to 12 Hz in 40 logarithmic steps. Given the study design and prior results (Smeds et al. 2017), we were especially interested in the activity at the first harmonic of the fundamental stimulation frequency (3.56 Hz), which was measured by setting one frequency bin to match exactly this frequency (Mujunen et al. 2021). In the design of the wavelet decomposition, we prioritized spectral resolution over temporal resolution (Delorme and Makeig 2004). As a result, the full width at half maximum accuracy of the transformation was 1.18 Hz and 740 ms at the response frequency band (Cohen 2019).

From the *t*–*f* representations of the responses (group averages presented in Supplementary Fig. S4), we could see the evoked EEG response to encompass strongly “steady–state-like” characteristics with the highest ITC values concentrating mainly within a narrow response frequency band. We wanted to quantify this activation by devising a channel-specific CKC metric. It was defined as an average of all ITC values of the *t*–*f* decomposition from the response frequency (3.56 Hz), excluding the values of the first and last 200 ms of the epoch length. CKC values range between 0 and 1 representing the overall magnitude of the elicited EEG activation time-locked to the stimulation.

Statistical significance related to a given ITC value was calculated using parametric circular statistics (e.g. Kabdebon et al. 2022). Rayleigh test evaluates the probability that a single coherence value results from a random phase distribution, i.e. phases uniformly distributed around a circle. Assuming that the phase distribution is unimodal and the data are sampled from the von Mises distribution, we used an equation (Berens 2009), which estimates the *P*-value as:

$$\begin{equation*} P={e}^{\sqrt{1+4N+4{N}^2\big(1-{ITC}^2\big)}-\big(1+2N\big)}, \end{equation*}$$

where *N* is the number of trials. The approximation is valid, provided the number of trials is sufficiently large (Cohen 2014). We determined the significance of the CKC by averaging all the *P*-values of the ITCs within the aforementioned *t*–*f* range. An alpha level of 0.01 was used as a threshold for statistically significant CKC response detection. When needed, the false discovery rate (FDR) correction for multiple comparisons was performed using the procedure by Benjamini and Hochberg (1995).

As seen from the equation above, in theory, the statistical significance of the ITC-based findings could be dependent on the amount of analyzed data and epoch division. In our analysis, we assessed the technical trade-off between recording length and reliability of response detection with a simulated test that repeated the CKC response detection using an increasing number of epochs included (taken cumulatively from beginning of the recordings).

### Influence of artifacts on the response detection performance

Neonatal EEG studies in the clinical environment are often performed in suboptimal recording conditions. Interference from external (e.g. electrical devices) or internal sources (e.g. respiration and cardiac activity) may couple to the EEG acquisition and results in signal artifacts and extensive noise (Keil et al. 2022). These technical adversities can be defeated only partially by an expert technician (preparation of electrodes and cables) and various post-processing techniques (filtering and signal decomposition tools). We wanted to test how our study paradigm copes with such situations using simulated test scenarios where authentic CKC recordings were deteriorated by adding various kinds of artifacts (as in Räsänen et al. 2013).

To reproduce the test scenarios as realistically as possible, the artifact signal components were extracted from previously recorded example EEGs known to be specifically contaminated by certain artifacts: i) electric muscular activity (EMG; frequency band 5–70 Hz), ii) electromagnetic interference caused by mains and external devices (2–125 Hz), iii) movement due to respiration (1–30 Hz), and iv) electric cardiac activity (ECG; 2–35 Hz). We used bandpass filtering to isolate the artifact from other signal components (cutoff frequencies above). Before superimposing them to the CKC recordings, the extracted signals were first normalized and then amplified by different gain factors to simulate different degrees of artifact coupling severity. After mixing the epochs of our original recordings with the artifact signals (summed together), the CKC response detection was repeated to estimate how much the given artifact type and magnitude affects the response rates.

### Network analysis using cortical source signals

The functional networks associated with the evoked CKC responses were analyzed at source space signals that provided improved spatial resolution and separation of cortical activities. The 21-channel EEG data were transformed into cortical parcel signals using source reconstruction described with details in our previous work (Tokariev et al. 2019). The transformation was composed of a realistic 3-shell (skull, scalp, and intracranial volume) head model segmented from an MRI of a full-term infant, a forward solution calculated with the symmetric boundary element method (Gramfort et al. 2010), and an inverse solution generated by dynamic statistical parametric mapping (Dale et al. 2000). Tissue conductivities of the segmented volumes were set to 0.20 S/m for skull, 0.43 S/m for scalp, and 1.79 S/m for cerebrospinal fluid. The procedure yielded 8,014 source signal components corresponding to fixed source space dipoles orientated orthogonally to the cortex surface. The source signals were clustered into 58 parcels symmetrically across the hemispheres and corresponding parcel signals were computed as weighted mean of their activity (Tokariev et al. 2019). Each parcel was categorized, based on its anatomical location, as frontal (F), central (C), temporal (T), or occipital (O).

We used directed PTE (dPTE) to evaluate functional connectivity and information flow between brain regions (Hillebrand et al. 2016). It is an established measure of directional connectivity calculated between pairs of phase time-series extracted from the cortical signals using Hilbert transform. To focus on the connectivity patterns specifically activated by the movement stimulation, an additional set of filters (high-pass and low-pass Hamming-window FIR filters, forward–backward, order 626) was applied before the Hilbert transform to constrain the signals tightly around the response frequency band (3.56 ± 0.3 Hz).

In the algorithm presented by Hillebrand et al. (2016), bidirectional PTE (Lobier et al. 2014) values between signals *x* and *y* (PTE_xy_ from *x* to *y* and PTE_yx_ from *y* to *x*) are normalized together to yield a measure of dPTE that estimates the preferred direction (“bias”) of the information flow:

$$\begin{equation*}{\text{dPTE}}_{xy}=\frac{{\text{PTE}}_{xy}}{{\text{PTE}}_{xy}+{\text{PTE}}_{yx}}.\end{equation*}$$

The dPTE values range between 0 and 1, with a value of 0.5 indicating equal causality between the signals. dPTEs were calculated between all parcel signal pairs, resulting in an individual 58 × 58 interaction matrix (like in Fig. 1D) that comprises the functional connectivity estimates (edges) between all the parcels (nodes).

Based on stimulations with our source reconstruction model, we knew that certain connections would have tendency to yield “noisy” connectivity measures due to suboptimal number and layout of recording electrodes (details in Tokariev et al. 2019). To improve reliability of further analyses, these edges were identified (32% of all possible connections) and removed from the interaction matrices using a binary fidelity mask common for all recordings.

### Consistent network

Directional connectivity data were analyzed both individually and at group level. In the group analysis, we aimed to distinguish the networks formed by the connections that emerged as the strongest consistently within the cohort (details of the procedure presented in Tokariev et al. 2019 and Ahtola et al. 2020). First, the 5% of edges with the highest dPTE values were selected from each individual interaction matrix. This proportion, later referred to as *k*-value, specifies the expected size of the network. The resulting binary arrays were summed over the group yielding a matrix that shows the strongest edges for a given stimulus condition at group level. Consistent network (CN) was determined statistically from this prevalence matrix as the subset of edges that survive an edge-by-edge binomial test (probability of success was set to 5% corresponding to the *k*-value, statistical alpha level to 0.05, and number of occurrences to 20 matching the number of infants). FDR correction (Benjamini and Hochberg 1995) was used to control for multiple comparisons.

### Spreading index

While calculation of CN provided us a proxy for response networks at group level, we needed another approach to compare the connectivity patterns between individuals. To this end, we devised a novel parameter called Spreading Index (SI) that aims to quantify the extent of the outbound-biased information flow from an individually selected subset of parcels that respond most prominently to the stimulation.

First, we created a reference distribution of surrogate dPTE values by pooling together all parcel pairs from the control recording of a single individual. Typically, these dPTEs were normally distributed around the average of 0.5. Next, we compared each dPTE_xy_ (element in 58 × 58 matrix) from the movement stimulation to the mean (μ) and standard deviation (σ) of the reference distribution and calculated a standard Z-score:

$$\begin{equation*}{z}_{xy}=\frac{{\text{dPTE}}_{xy}-\mu}{\sigma}\end{equation*}$$

The *Z*-scores can be used to distinguish connections that are significantly different from the mean of the surrogate data at a given alpha level using a right-tailed *Z*-test. We determined individually a subset of 4 parcels, “source nodes,” that generated the highest number of significant outbound edges (*P* < 0.01) from the contralateral hemisphere and, finally, calculated the proportion between the number of significant edges and all possible edges originating from these sources. The resulting percentage is the SI that ranges between 0 and 100% and reflects the extent of information spread from the primary source nodes to secondary areas. Similarly to CKC calculations, SI can also be extracted from the ipsilateral response components by constraining the source node selection to the ipsilateral hemisphere (in relation to the stimulation side). This was used in the evaluation of the response laterality (see below).

### Symmetry of the response

Since movement stimulation was performed separately for the right and left hands, the responses were also asymmetric and allowed obtaining separate metrics for each stimulation side. Hence, we could also assess *symmetry* of the response as a ratio (0%–100%) between the lower and higher scoring sides. This was hypothesized to be affected by unilateral brain damage.

We also wanted to assess hemispheric lateralization of the response with respect to stimulation side. This *laterality* metric was derived from a combination of the contralateral and ipsilateral values for CKC and SI metrics and expressed as a ratio (0%–100%) between the contralateral value and the sum of the contralateral and ipsilateral values. Laterality of 100% indicates that the response is completely contralateral, whereas 50% implies equal response in both hemispheres after unilateral stimulation.

### Statistics

The pairwise group comparisons (based on clinical gradings, MRI findings, or vigilance states) of continuous variables (such as CKC metrics) were carried out using Mann–Whitney *U* test with an alpha-level of 0.05. A more conservative alpha level of 0.01 was chosen for the detection of CKC responses. A FDR correction (Benjamini and Hochberg 1995) was applied to control the family-wise error rate stemming from the multichannel EEG data (q-parameter set to 0.01).

### Analysis software

All signal processing and analyses in this work were performed using custom-scripted MATLAB routines (version R2018A) and functions within several freely available MATLAB toolboxes.

EEG was preprocessed using FieldTrip toolbox (Oostenveld et al. 2011, https://www.fieldtriptoolbox.org) and transformed to CSD signals using functions of CSDtoolbox (Kayser and Tenke 2006a, 2006b, https://psychophysiology.cpmc.columbia.edu/software/csdtoolbox). For the source reconstruction, we used openMEEG toolbox (Gramfort et al. 2010, https://openmeeg.github.io) and Brainstorm toolbox (Tadel et al. 2011, https://neuroimage.usc.edu/brainstorm). In connectivity analyses, dPTE was calculated using a function “PhaseTE_MF” included in Brainstorm Toolbox (Tadel et al. 2011). The version we used was 2.5 (June 2017) with the default approach “scott” applied for the calculation of bin size for phase occurrence histograms. CKC responses were visualized in topographic 2D views with EEGLAB toolbox (Delorme and Makeig 2004, https://sccn.ucsd.edu/eeglab/index.php) using the inverse distance interpolation method, while their statistical significance was evaluated using a function “circ_rtest” from CircStat Toolbox (Berens 2009, https://www.jstatsoft.org/article/view/v031i10).

The function for computation of CNs is freely available online for download at https://github.com/babyEEG/neoNets. The novel codes for detection of CKC responses and calculation of related cortical networks and SI metrics are all shared at https://github.com/ahtolee/CKC-EEG-Networks, along with compatible example data.

## Results

### CKC responses were detected from all recordings

**Fig. 2:**
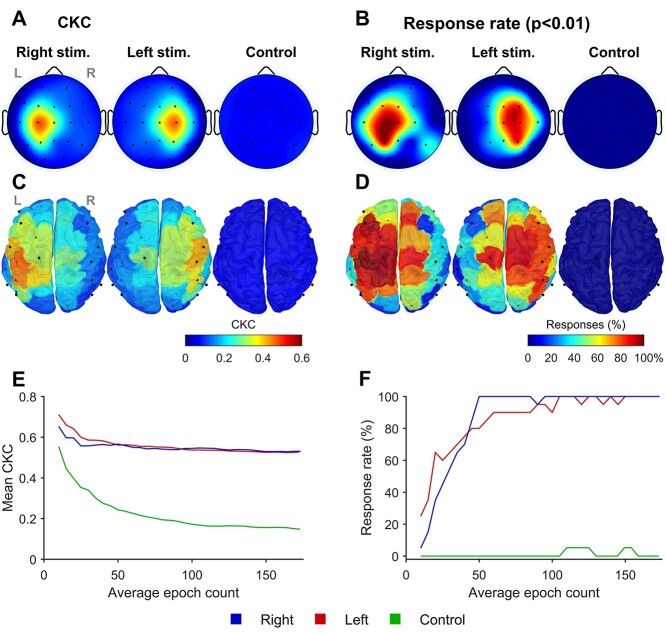
CKC response detection. Topographic head plots in (A) show group averages of CKC values calculated from the scalp EEG recordings of the 3 stimulus conditions. B) Corresponding channel-specific response rates with the statistical threshold set to *P* < 0.01. C) and D) Corresponding CKC and response rate distributions that were calculated from cortical parcel signals and then projected over a 3D cortex model. E) and F) The peak CKC magnitudes (averaged over the cohort) and corresponding response rates (FDR applied) as a function of the amount of data. Note the ceiling effect in response rate between 50 and 100 epochs (F), while no change is seen in the mean CKC after about 40 epochs (E).

For the movement stimulation, significant CKC responses (*P* < 0.01) were detected from both hands in all subjects (Fig. 2B). For the control stimulation without physical contact to the participant, no significant responses (e.g. auditory ones) were detected in any of the infants. Analyses of the sensor space data (CSD montage) indicated clearly contralateral responses with the highest CKC observed at the C3/C4 electrode locations (Fig. 2A) that correspond to the sensorimotor cortices (see also Smeds et al. 2017). Individual-level results for all infants are shown in Supplementary Fig. S1.

For a comparison, we performed also cortical level CKC response analysis with the same data transformed into parcel signals, which showed more varying and widespread cortical responses (Fig. 2C and D). Like the sensor space analyses, cortical responses were also strongest (Fig. 2C) and most prevalent (Fig. 2D) at the parietal lobe of the contralateral cortex. Yet, the responses were also frequently detected at many other nearby cortical regions, including an ipsilateral component in many infants (cf. Bourguignon et al. 2012; Vallinoja et al. 2021).

We also assessed how the response detection is affected by the recording length and found that increasing the number of epochs included in the CKC analysis resulted in a ceiling effect: the peak CKC magnitude (averaged over the cohort) plateaued after about 40 epochs, equaling 44 s of stimulation (Fig. 2E). Conversely, the proportion of infants showing a significant CKC response (*P* < 0.01) increased with the epoch count until a 100% ceiling was reached after 50–100 epochs (55–110 s). Increasing the amount of data did not bring artificial CKC responses with the control stimulation, though there was always some nominal CKC value observed in the control condition as well (Fig. 2E and F).

In order to verify that the results are not too dependent on the choice of the computational method, we also examined the CKC responses with other signal processing techniques, such as calculating the phase coherence at the response frequency in spectral domain using fast Fourier transform (as in Kabdebon et al. 2022). The findings were highly comparable with the wavelet-based algorithm primarily used in this work, which was expected from the fundamental similarities in the decomposition methods. A correlation between CKC values from the 2 methods was as high as *r* = 0.97 (Pearson’s test). However, the wavelet-based method was found to give a better signal-to-noise ratio with on average 14% higher peak CKC values.

### Responses survive significant real-world artifacts

Our findings above show that the CKC responses are readily detected in all infants in well-controlled laboratory conditions such as those in our clinical research center. However, the practical utility of the method is potentially challenged by the real-world artifacts that often contaminate EEG studies in different recording environments. To assess the robustness of our method in suboptimal conditions, we conducted additional experiments by artificially adding artifact noise to our relatively clean data and subsequently testing the algorithm’s capability to sustain successful CKC detections.

**Fig. 3:**
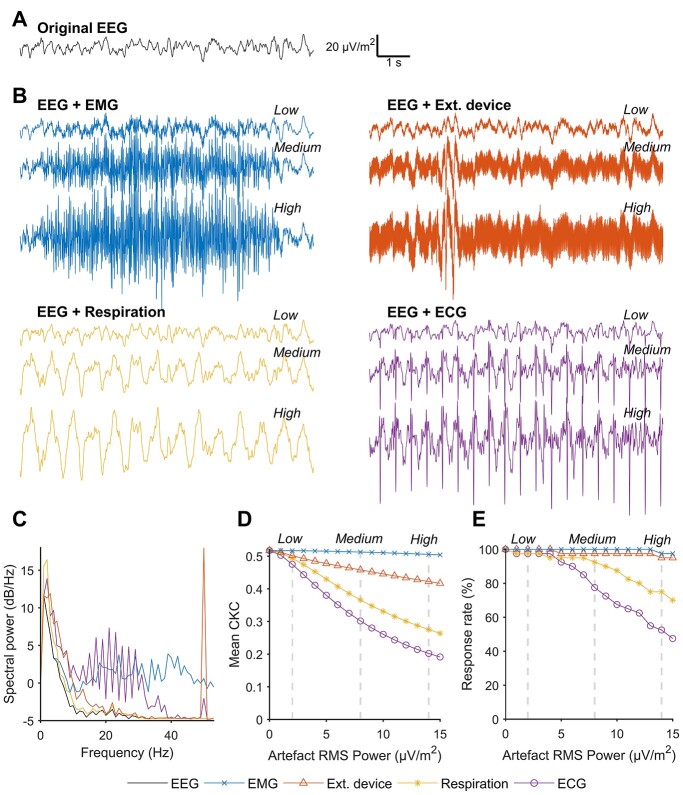
Influence of artifacts on the response detection. A) Example of a CSD-transformed electroencephalography signal (EEG, uncontaminated). B) Samples from the 4 artifact types that were superimposed to the CKC recordings, in this case, the signal of A. Three different severity levels (RMS power) of the artifact coupling are illustrated (“low,” “medium,” and “high”). C) Power spectral density graphs of the normalized artifact signals and the uncontaminated EEG signal (black). Panels (D) and (E) show how the group average CKC (individual maxima) and response rates (*P* < 0.01) deteriorate if the artifact magnitude is increased. The response detection copes particularly well with EMG and device artifacts but suffers from respiration and ECG artifacts that comprise lower frequency components.

Examples of these contaminated data epochs and their spectral density graphs are presented in Fig. 3. Comparison of the frequency spectra of the artifacts shows that most of the artifact-specific power (i.e. what departs from the universal 1/f form) is localized at the frequencies that are much higher than the CKC response frequency (3.56 Hz). Only respiration frequency as such, as well as some components of the ECG waveform, may overlap the CKC response band.

As expected, sensitivity of the detection algorithm to artifacts was dependent on their spectral contents. Adding incremental amounts of artifact noise showed that both the mean CKC magnitude and the CKC response detection rate may survive even through the strongest tested EMG and electrical interference levels. The CKC response rate was also found to survive moderate levels of respiration and ECG artifacts; however, the mean CKC levels were gradually decreased with the increasing power of these artifact types in particular. From the 4 tested scenarios, the spiky ECG artifact poses the greatest threat for the algorithm. In the most severe (“high”) ECG artifact condition (RMS power 14 μV/m^2^), the response rate drops to 50% (Fig. 3E), but at this point the deteriorated EEG would already be practically “unreadable” due to the predominant artifact component (Fig. 3B).

We also validated the original response detection (cf. Fig. 2A and B) by replacing the manual artifact screening with an automatic artifact rejection procedure based on simple amplitude thresholding instead. This yielded nearly identical results (data not presented) showing significant CKC responses (*P* < 0.01) from both hands in all infants, while no responses were detected from the control condition. Although visual inspection of the recordings is always recommended, these results indicate that the preprocessing steps in the analysis pipeline could be easily automatized.

### CKC activates cortical response networks

Analysis of cortical activity time-locked to the stimulation revealed CKC responses over wide-ranging cortical areas (Fig. 2C and D and Supplementary Fig. S1). As expected, some responding parcels were also frequently (68% of recordings) found in the ipsilateral hemisphere. To probe the CKC-related connectivity patterns, we assessed direction of the information flow between the parcels by calculating dPTE between pairs of bandpass filtered cortical signals.

**Fig. 4:**
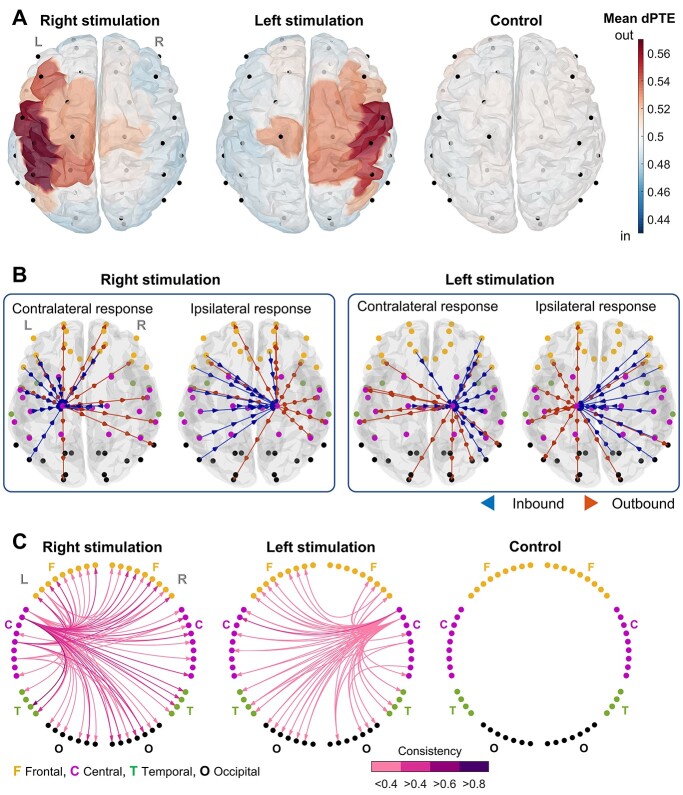
Response networks. A) Group averages of the nodal dPTE values. dPTE >0.5 (red) means that the amount of nodal outbound information flow exceeds the corresponding nodal inbound flow. B) Strongest 10 inbound and outbound connections of symmetrically chosen contralateral and ipsilateral parcels that were generally outbound biased. The graphs illustrate the difference of connectivity patterns between the hemispheres: The contralateral parcel receives information flow mainly within the hemisphere and the ipsilateral parcel across them. C) CN graphs (*P* < 0.05, *k* = 0.05), as circular diagrams, include the connections that appeared consistently among the strongest across subjects. The color scale of the arrows depicts the proportion of infants showing the given edge (i.e. “consistency”). The connections measured during the control stimulation were highly random and hence did not yield any consistent pattern.

Figure 4A presents parcel-by-parcel (i.e. nodal) group averages of the dPTE values projected over the 3D cortex model. Averaging shows the net bias between inbound and outbound information flows to/from a network node revealing whether the node operates mainly as a source transmitting information or a sink receiving it. Notably, the topographical distribution of the most outbound dPTE values is remarkably similar to the CKC pattern presented in Fig. 2C. These findings are compatible with a view that these peak parcels represent the primary somatosensory cortex, which will be the primary source of outgoing (red) information flow to drive the CKC response over to other cortical regions. On the contrary, we could not distinguish any distinct parcels that would stand out as information sinks. At group level, the information is distributed from the primary sources across the cortex quite evenly.

As shown in Fig. 4A, a single parcel was found to stand out as an outgoing source of information flow in each ipsilateral hemisphere. We next wanted to examine its relationship to the anatomically corresponding parcel on the hemisphere contralateral to the stimulation. We examined the strongest 10 inbound and outbound connections associated with these 2 nodes (Fig. 4B) and found that this ipsilateral parcel mostly receives information from the contralateral side and distribute it further to other ipsilateral areas. The net outbound flow in this parcel exceeds its inbound flow; hence the node appears more as an information source than a sink (dPTE >0.5).

While the average primary source areas seemed to converge nicely to consistent neuroanatomical rationales, there was substantial inter-individual variation in the overall level of dPTE strengths in the networks (Supplementary Fig. S1). Such variation supports reduction of network complexity and data variability by estimating group-level CNs (Tokariev et al. 2019; Ahtola et al. 2020) based on statistical comparison of the subsets of the strongest edges in the recordings (group averages also presented in Supplementary Fig. S5). The CN diagrams presented in Fig. 4C (per stimulus condition) were calculated by selecting the strongest 5% of edges in each interaction matrix (*k*-value) and setting the statistical threshold to *P* < 0.05. In line with the findings above, we found 3–4 contralateral parcels operating as the primary sources in the group-level CKC networks, distributing information flow within and across the hemispheres. Notably, the CN patterns were highly symmetric between sides of stimulation, while the control (auditory) stimulation failed to elicit any significant CNs. Corresponding network graphs with different *k*-values (expected network size) and alpha levels are presented in Supplementary Fig. S6.

Additional analyses of the CKC networks are presented in Supplementary Fig. S7 where we examined edges that were found to significantly differ from the reference data (control recordings) based on *Z*-test statistics. We observed that while the number of the most active source parcels was generally constrained, their locations varied between individuals. This information was used in the development of the SI that we applied in the subsequent individual-level analyses where the effect of vigilance state and clinical condition were evaluated.

### Effects of vigilance states and severity of neurological adversity

We finally wanted to examine how the CKC is affected by the clinical key scenarios. Vigilance state is the behavioral context in all assessment, while neurological problems caused by varying degrees of brain injury are the clinical context in most studies in human infants.

### Vigilance state

**Fig. 5:**
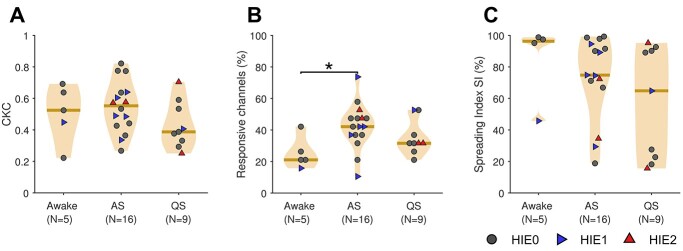
Responses and network metrics of recordings performed during different vigilance states: awake, AS, and QS. A) CKC magnitude defined as channel-specific maximum. B) Response coverage defined as the percentage of channels that showed a significant (*P* < 0.01) CKC response. C) SI reflecting the extent of information flow from the primary source nodes to secondary areas. Panels A) and B) were calculated from the scalp data and (C) from the parcel source signals. Results from right and left hand stimulations were pooled together in all graphs, while the different markers of the recordings depict infants in different clinical subgroups (HIE0, HIE1, HIE2). The horizontal lines represent the median values of the distributions. The asterisk symbol between the response coverages in Awake and AS conditions implies a statistically significant (*P* < 0.05) difference.

As newborn babies sleep daily between 18 and 22 h, most neurophysiological neonatal studies are carried out during sleep. Hence, it is essential to estimate the effect of vigilance state on the results (Pihko and Lauronen 2004). In our data, the level of CKC, measured at the channel maximum, was not significantly affected by the prevailing vigilance state (Fig. 5A), although the CKC response appeared slightly weaker during QS. However, the spatial extent of CKC responses was significantly larger in AS than awake (42% vs. 21% of CSD channels; *P* < 0.05; Mann–Whitney *U* test; Fig. 5B). The SI, i.e. the extent of cortico-cortical information flow from the primary somatosensory cortex, however, was not significantly affected. Plotting the diagnostic subgroups into the graphs (note the marker symbols) shows that there was no apparent systematic bias to explain the observations.

### Severity of perinatal asphyxia

**Fig. 6:**
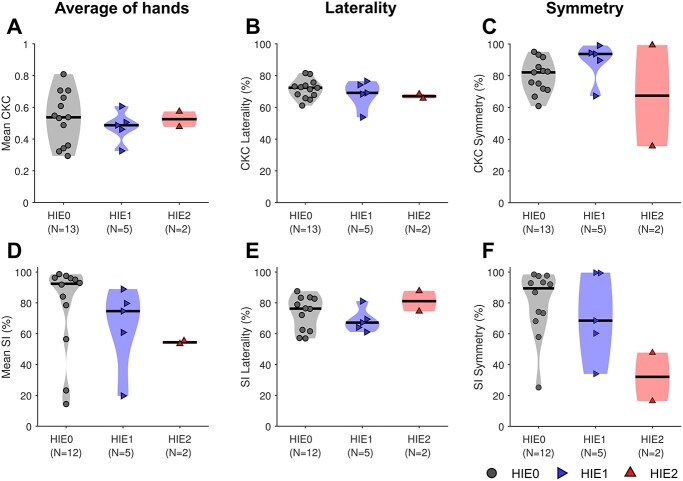
CKC metrics of infants in different clinical subgroups (HIE0, HIE1, HIE2). Panels (A)–(C) depict response magnitudes measured with CKC, whereas (D)–(F) show SI values that quantify the degree of information flow in the networks activated by the stimulation. CKC and SI were assessed based on their average magnitude, mean laterality, and symmetry between right and left side stimulations. The horizontal lines represent the median values of the distributions.

As a tentative evaluation of the clinical potential of the presented method, we compared response and network metrics between the subgroups of infants with different clinical conditions: perinatal asphyxia without HIE (HIE0), mild HIE (HIE1), and moderate HIE (HIE2). Figure 6 presents mean (of right and left hand) CKC and SI as well as their average laterality and symmetry for the infants in these subgroups. For one infant (HIE0), the SI metrics could not be computed due to missing control recording.

The differences observed in the comparisons were often subtle. Measured with CKC, the response magnitudes were typically high for stimulations of both sides. CKC symmetry was above 60% in HIE0 and HIE1 groups, but one HIE2 case produced symmetry of less than 40%, i.e. the CKC response magnitude of right hand stimulation was less than half of the left side. CKC laterality tends to decrease for infants with higher HIE severity.

The same propensity was also present in the comparisons of SI distributions, where the overall variation was greater. On average, SI decreased in more severe clinical subgroups (HIE1, HIE2). Although the average SI in HIE0 and HIE1 groups was 77% and 65% respectively, there were also 3 clear outliers, i.e. infants with constrained information flow in the networks activated by the stimulation (Fig. 6D). The 2 cases with HIE2 yielded low SIs for the right hand stimulation (compared with the left), which resulted in highly biased SI symmetry (Fig. 6F). Yet, the size of the HIE2 group does not permit reliable statistical evaluation. Moreover, there were no significant differences in SI laterality values between the 3 groups.

### Structural brain injury

The abnormal brain MRI findings were further categorized into white matter or gray matter findings. There was substantial variation in CKC and network metrics in the infants with no WM findings, and thereby no correlation was observed between these metrics and grade of the WM findings (data not shown). To simplify the statistical comparison, the infants were divided into 2 subgroups based on whether there were findings of either type (GM or WM) in their MRIs. CKC and network metrics of the infants in these groups are presented in Supplementary Fig. S8. It appears that infants with MRI abnormalities (*N* = 9; one infant omitted due to missing control recording) show a tendency to yield slightly lower and more asymmetric SI values compared with those with normal MRI (*N* = 10). Yet, neither of these observations were statistically significant within the studied cohort. Analysis of the CKC response magnitude did not reveal considerable differences between the groups.

## Discussion

We showed that cortical networks can be recruited reliably in awake and sleeping newborn infants by using a pneumatic stimulator that generates passive, repetitive hand movements. These responses can be quantified by CKC measured from the scalp EEG signals. The recruited functional networks were highly consistent across the infant cohort that varied with clinical presentations and vigilance states. The findings were in line with previous evidence (Smeds et al. 2017; Bourguignon et al. 2019), showing that the CKC response originates mainly in the primary sensorimotor cortex and then propagates to nearby cortical areas related to motor functions, eventually covering wide cortical regions. Our findings show that the extent of CKC spread might be affected by neurological conditions, such as perinatal asphyxia and/or hypoxic–ischemic encephalopathy. The results support the use cortical CKC after pneumatic hand movements as a scientific and/or clinical tool for a controlled assessment of cortico-cortical networks related to sensorimotor integration.

This study aimed to develop a clinically applicable, end-to-end solution for assessing cortical networks involved in somatosensory processing. To this end, we developed an automated, naturalistic stimulation method with a pneumatic pump and constructed an analytic pipeline for assessing cortical CKC responses and cortico-cortical recruitment from the EEG signals; thereafter, we evaluated reproducibility and clinical usability of the developed method with term neonates having perinatal asphyxia. CKC is considered to arise from neuronal communication between cortex and peripheral proprioceptors (Proske and Gandevia 2012); in addition, it is likely that CKC responses after passive hand movements also involve peripheral tactile input, and perhaps a mixture of cortical and subcortical inputs (Piitulainen et al. 2013; Bourguignon et al. 2015, 2019).

Passive stimulation is generally useful when aiming to separate sensory and motor systems. In the infant studies, however, passive stimulation is mandatory due to lacking cooperation. Here, we used a custom-built movement actuator, which yielded significant (*P* < 0.01) CKC responses for both hands in all (100%) infants. No false detections were observed (responses in the absence of physical stimulation) suggesting high sensitivity and specificity. The CKC responses were even resistant to incremental addition of various sources of naturalistic noise in the signal; only substantial amount of low-frequency noise (e.g. respiration and ECG interference) had notable deteriorating effect on the response detection. Hence, the automated movement stimulation and its associated analytic pipeline appear to provide added sensitivity and robustness compared to the previously published manual stimulation (Smeds et al. 2017) where 3 out of 13 studied infants showed only partial CKC responses.

The strongest CKC was generated at the first harmonic of the fundamental movement frequency (at 3.56 Hz), i.e. frequency associated with flexion–extension phases of the movement stimulus. The same was observed also previously in infants (Smeds et al. 2017) but less systematically in adults where the fundamental and the first harmonic frequencies show more equal CKC strength (Piitulainen et al. 2013, 2015, 2020). The CKC responses peak near contralateral primary sensorimotor cortex; however, the parcels with strongest CKC were located slightly more lateral compared to the parcels that showed highest likelihood (response rate) of a significant CKC response. Nevertheless, the results showed clearly that CKC is not confined to a single cortical spot; it rather encompasses wider cortical areas, including midline and the ipsilateral hemisphere that is also known to receive direct proprioceptive input from the periphery. The variation between individual topographic maps may be related to immature organization of the newborn cerebral cortex (Nevalainen et al. 2014) and/or to the unavoidable variation of electrode placements in respect to the cortical areas (Kabdebon et al. 2014).

Despite the variation in local CKC response topographies, there was a salient inter-individual consistency in the cortical networks associated with CKC response. We could show how the most active contralateral Rolandic areas, in terms of processing somatosensory afference, operated as the driving sources distributing the information flow to other cortical regions (Fig. 4C). The analysis of network patterns also depicted 2 symmetrically located parcels that convey CKC-related information flow to the ipsilateral areas (Fig. 4B), possibly operating as “network relays” between the hemispheres. Although some of the CKC-related processing is likely emerging through the direct thalamocortical connections (Nevalainen et al. 2014), it is reasonable to assume that the full cortical extent of the CKC response arises largely as a cortico-cortical spreading from the primary somatosensory cortex. Hence, we suggest that both CKC magnitude and network characteristics could be used as clinically relevant parameters to assess cortical somatosensory function and recruitment of the related cortico-cortical networks in a controlled manner.

In addition to the “core network” that was consistently found in the infants, there were also other cortical connections that showed much higher inter-individual variability, both in their density and distribution. This motivated us devising metrics, such as SI, for measuring the network involvement. Previous studies have shown that the cortical extent of conventional somatosensory responses of neonates (e.g. Rahkonen et al. 2013; Nevalainen et al. 2017) may correlate with clinical outcomes. Our present findings provide tentative data that an increasingly severe brain injury (HIE) may link to less cortical spreading as well as less symmetric responses. Likewise, infants with abnormal MRI findings in their white and/or gray matter showed a tendency for lower SI and less symmetric CKC network. While physiologically plausible, these findings did not reach statistical significance in our small cohort, and more recordings, especially to infants with HIE, are needed to confirm these findings.

The exact mechanistic underpinnings of the development of CKC responses and their cortico-cortical spread are as yet poorly understood at the cellular level. However, related studies on animal models have shown that higher-order somatosensory cortical areas become responses to thalamic sensory input at the end of the first postnatal week (Cai et al. 2022). This suggests that the cortico-cortical mechanisms underlying CKC response are present at around full-term age in the human infants. Future studies are warranted to show the developmental changes in CKC responses, and their cortical spreading, during both preterm period and during infancy as a part of neurodevelopmental assessment, as well as in several other clinically relevant neurological conditions.

There was also a perhaps surprising spread of CKC responses to the parietal regions, which is likely due to both physiological and physical reasons. First, early brain development is characterized by highly exuberant cortical connections (Innocenti and Price 2005), and it may be possible to see cortico-cortical spread in the young infants to areas that become “disconnected” by pruning of connections later in life. Second, we cannot know exactly the spatial relation of our scalp electrodes and source activities to the functional cortical parcels. Therefore, some apparent spread between nearby regions, such as anterior parietal area, may be (see Bastos and Schoffelen 2016) due to physical reason (spatial smear) or technical reason (computational leakage). These can be fully disambiguated by combining very high-density recordings (over 100 electrodes; Yrjölä et al. 2022), digitization of the electrode coordinates, and individual MR imaging; such work is doable in centers where such methodology is available for research use in the human infants.

There are some technical considerations that merit attention when comparing our present findings to prior literature. Our present use of pneumatic stimulator called for streamlining the technical setup from the previous studies (e.g. Bourguignon et al. 2011; Piitulainen et al. 2013; Smeds et al. 2017), and we calculated CKC directly from trial-to-trial phase using only EEG signals without accelerometer data. Such solution comes at a cost of losing sensitivity to variations in movement frequency (Cohen 2014); it is not a problem when the stimulator operates at a stable frequency, which is also known to elicit stronger CKC than irregular stimulation (Mujunen et al. 2021).

The present analysis algorithm was built using freely available MATLAB toolboxes, and it can be compiled as an automatic (or semi-automatic) workflow that preprocesses the data and calculates the response graphs and associated *P*-values from a bedside-recorded EEG file in minutes. Our in-house trials have shown that such automated pipeline will may provide an easy access to objective and consistent studies of somatosensory function, which is a significant advance compared to the conventional clinical performance and interpretation of evoked potentials via averaged trials in the time domain. This pipeline may also be optimized, for instance by focusing only on the spectral component of the frequency of interest rather than producing the full time- and frequency-resolved representation presented in this work to provide further “quality control” at the level of single subjects and electrodes. One computationally suitable option is replacing the wavelet decomposition with Fourier-based analyses (Kabdebon et al. 2022).

In this work, we used a custom-built movement stimulator (see also Jousmäki 2021), which may hinder the uptake of the method. However, the stimulator solution needed for CKC recording is mechanically simple. We are currently piloting alternative options, such as commercially available electric breast pumps, which generate very comparable periodic suction, and they can be easily changed to movement stimulators. Our first pilot recording (Supplementary Fig. S9) showed that such application needs only low-tech level instrumentation that could be readily accomplished as a part of future clinical studies.

The insensitivity to both sleep state changes and real-life artifacts indicate general robustness of CKC as a measure of cortical function which can be applied in a wide range of recording environments and situations. Indeed, the hereby presented CKC paradigm holds potential for clinical research use as a completely noninvasive tool for a controlled assessment of cortical functions. CKC metrics could readily provide complementary information to the diagnostic routines of neurological examination, brain imaging, and EEG monitoring. Most importantly, consistent and reliable CKC responses can be elicited during a routine EEG study using naturalistic movement stimulation, and the computational analysis pipeline can be fully automatized, delivering statistically assessed study results; this is possible even in the presence of considerable noise that typically compromises all computational and visual EEG analyses. A particularly attractive research application of a method of this kind is perhaps early prediction of neurocognitive development (cf. Nevalainen et al. 2015, 2017) after mild-to-moderate degree brain injury during perinatal period. While outcome prediction is well established after a severe brain injury, there is scarcity of methods available for predicting outcomes in mild-to-moderate cases, which however are much more prevalent and hence clinically meaningful. It is conceivable that assessing functional integrity of cortical networks might provide a needed insight about cortical recovery potential after early adversity. However, prospective studies with larger patient populations are needed to examine this idea and to establish the perceived added value of CKC paradigm at bedside.

## Supplementary Material

Online_Supplement_bhac373

CKC-hand_stimulation_bhac373
