# Supplementary material for: Cortical networks show characteristic recruitment patterns after somatosensory stimulation by pneumatically evoked repetitive hand movements in newborn infants
Source: Cereb Cortex. 2022 Nov 11;33(8):4699–713. doi: 10.1093/cercor/bhac373 (PMC10110426; doi:10.1093/cercor/bhac373)
Supplement: Online_Supplement_bhac373 [file online_supplement_bhac373.zip › Online_Supplement_bhac373.docx]

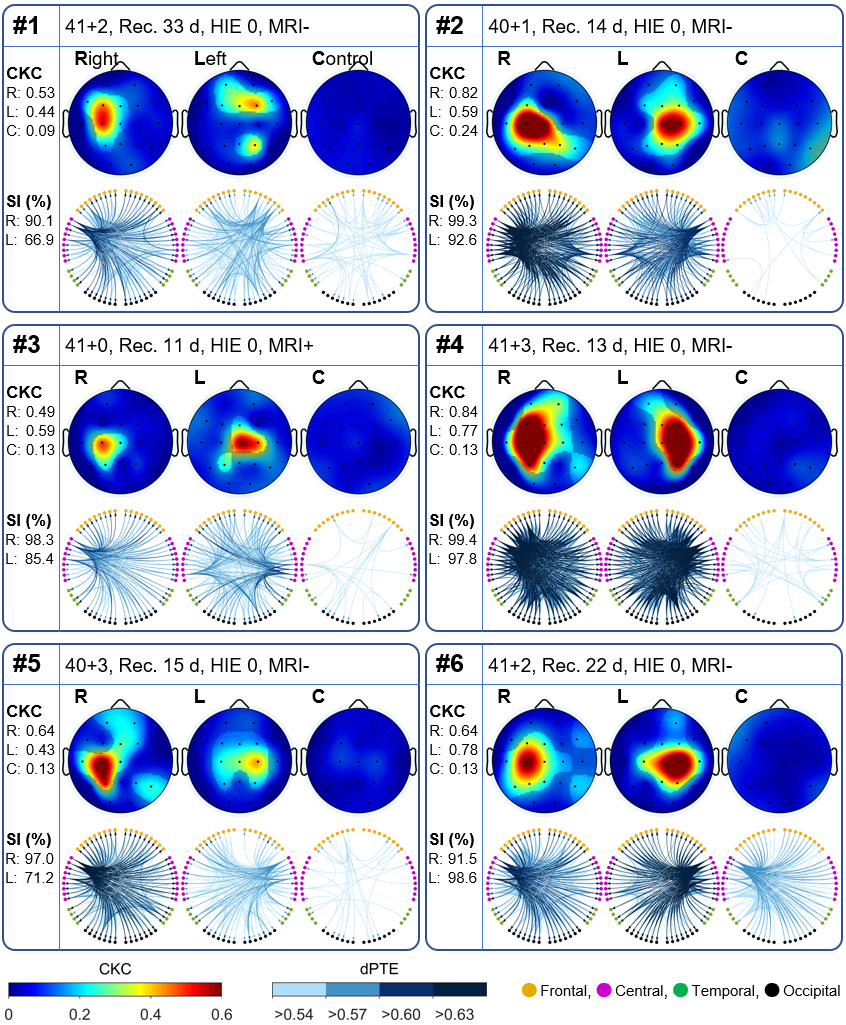


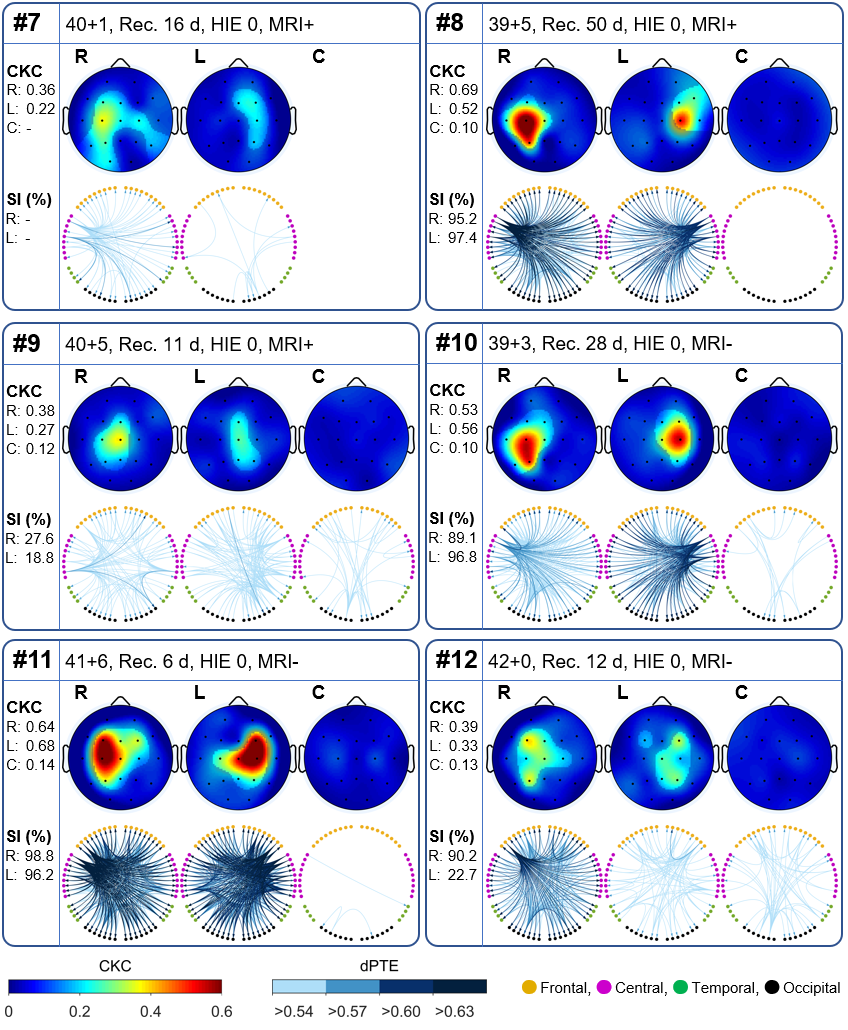


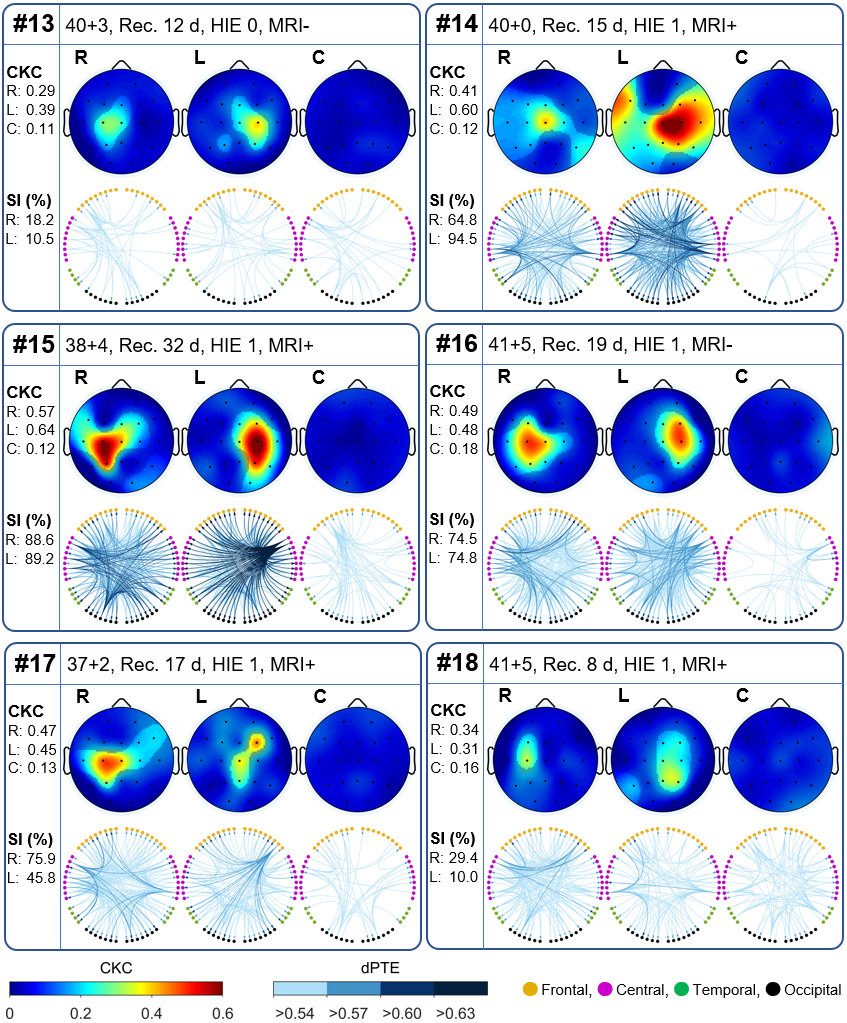


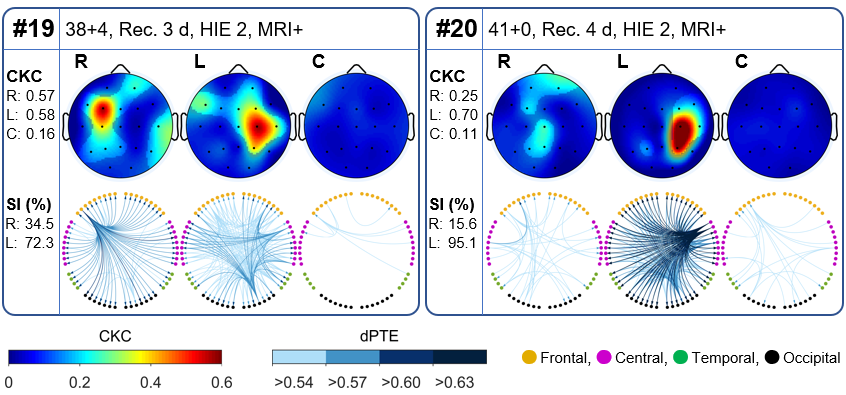


***Figure S1****. Individual results and clinical background. Each figure plate depicts corticokinematic coherence (CKC) response detection and directed phase transfer entropy (dPTE) network graphs from the recordings (R = Right, L = Left, C = Control) of one infant (#1–20). Control recording could not be performed for infant #7 due to technical challenges. Text panel above the graphs contains gestational age (weeks + days) and postnatal age at the recording (Rec. days), as well as clinical grade for hypoxic-ischemic encephalopathy (HIE: 0–3), and presence of magnetic resonance imaging findings (MRI: +/-).*

*A statistical threshold of p < 0.01 is applied as a semi-transparent mask on top of each CKC graph highlighting the topography of a significant response. The highest CKC value of the recorded channels is given on the information panel (left) and is thought to represent the peak magnitude of the elicited CKC. The response networks are presented as circular connectivity diagrams. All edges with dPTE above 0.54 (arbitrary visualization threshold) are illustrated with a blue colour scale that depicts the bias of the outbound information flow. The associated Spreading Index values (SI, shown on the left) measure the extent of information flow from the primary source nodes to the secondary areas.*


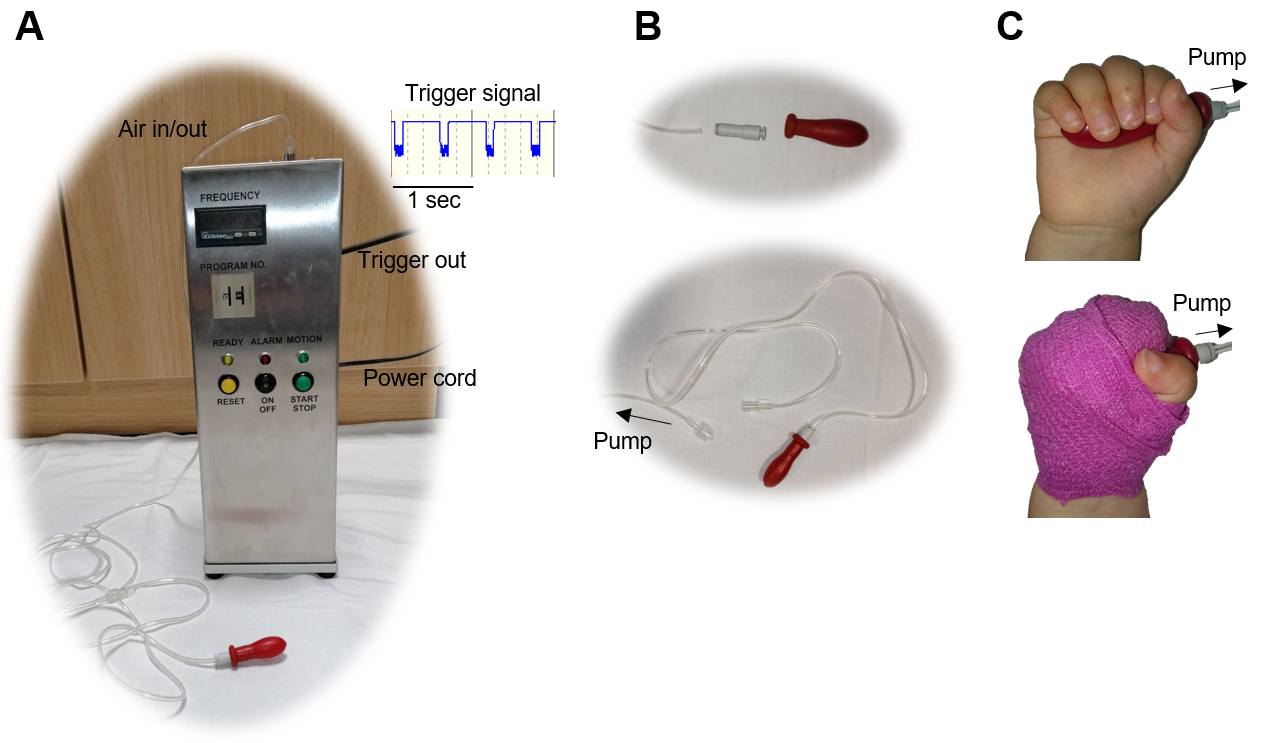


***Figure S2****. Setup of the hand movement stimulator. A pneumatic pump (A) generates periodic inflate/deflate cycles of a pipet bulb via plastic tubes (B) and transmits an analogue trigger signal to the EEG recording. The bulb is wrapped inside the infant’s palm (C) to actuate passive finger extensions of the hand. The stimulator pump was stored in an aluminium case (not shown in the picture) to insulate the operation noise.*


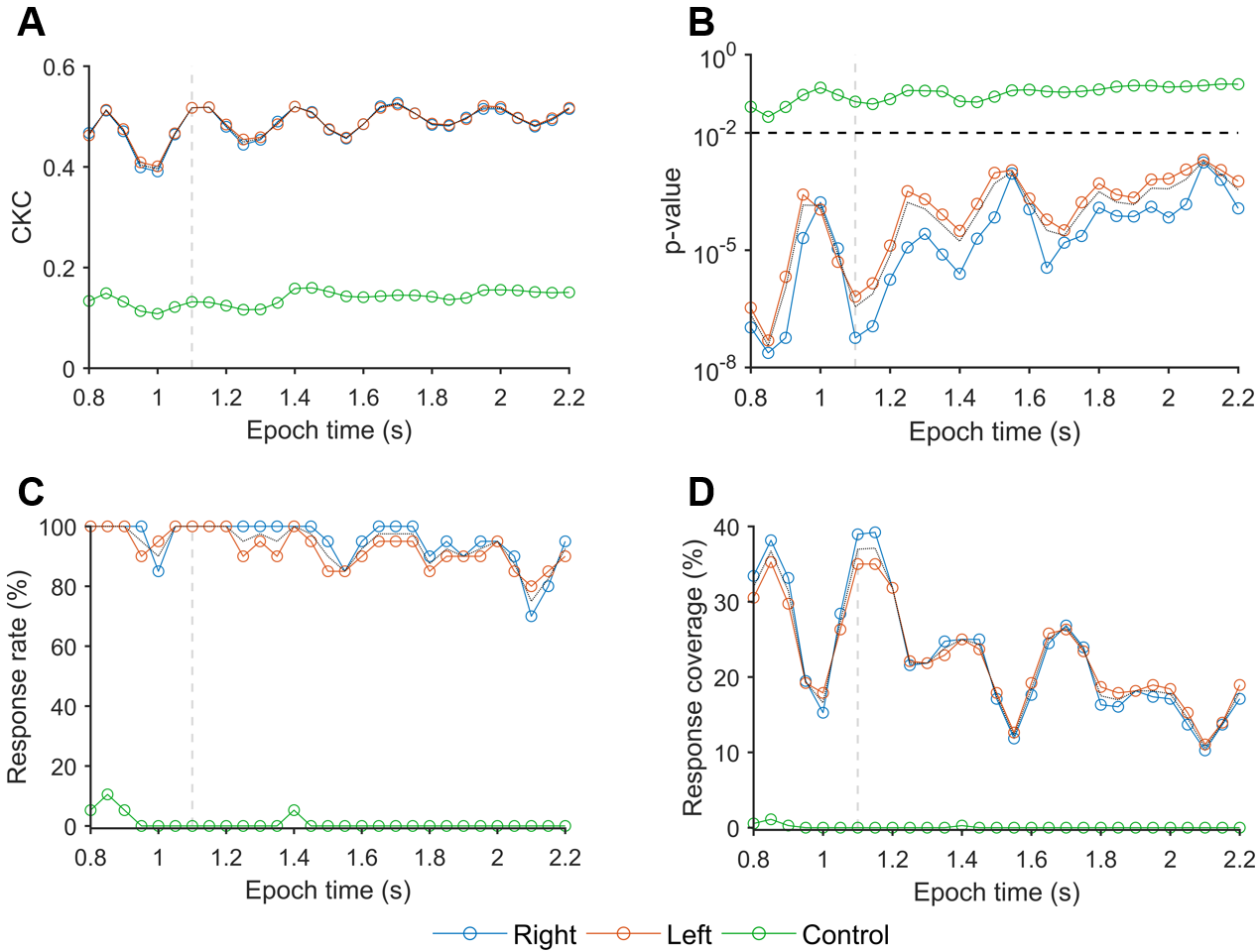


***Figure S3.*** *Response detection with different epoch times. Graphs show corticokinematic coherence (CKC) response detection data gathered after experimenting with different epoch durations used in the epoch division. Epoch times between 800 ms and 2200 ms were tested (200 ms pre-stimulus interval included) in 50 ms increments. Consecutive epochs were not allowed to overlap in the division, while segments with notable artefacts were always discarded.*

*In general, the detection algorithm seems quite robust for changes in the epoch division. Averaged CKC values (A) remain stable with only minor periodic variation emerging from the proportion between the interstimulus interval (561 ms) and the epoch length. If these intervals do no match optimally, pieces of the data are always lost in the segmentation. The same periodicity can be observed from the corresponding statistical p-values (B). Additionally, they showed a tendency to slightly increase when longer epoch times were applied, and total number of epochs consequently decreased. Applying alpha level of 0.01 as a threshold for statistical significance (marked with horizontal line in B) resulted in marginally reduced responses rates (C) and smaller response coverage in general (D; proportion of channels that show significant CKC).*

*Based on this data, we chose to use epoch length of 1100 ms in our analysis (marked with vertical grey line), as it produced typically the lowest p-values, the highest response rates, and most extensive coverage.*


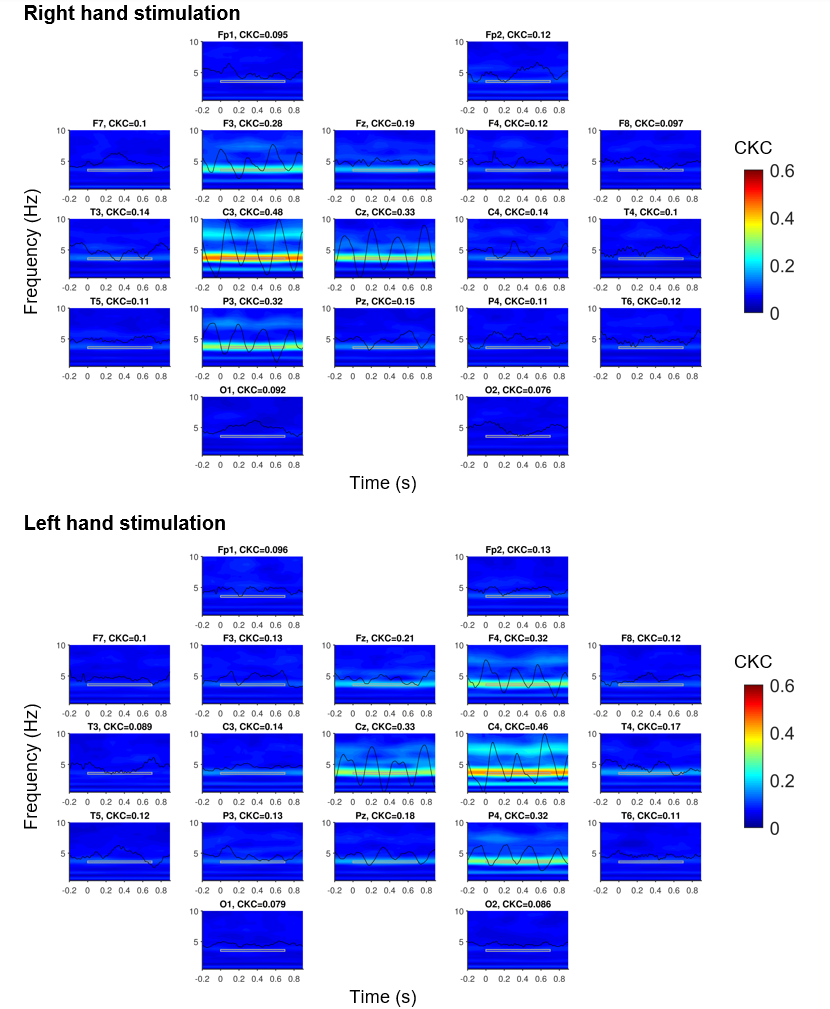


***Figure S4****. Group averages of the inter-trial phase coherence (ITC) time–frequency representations for right and left hand stimulations. Averaged and normalised event related potentials (ERP) are projected over each subplot, but they were not used in the response analysis. Corticokinematic coherence (CKC) metric for each channel was calculated by averaging ITC values from a narrow response frequency band that matches the first harmonic of the fundamental stimulation frequency (3.56 Hz). This area is depicted as a grey rectangle on top of the graphs, and the result is given in the title of the subplot (group average).*

*The graphs show that at group level the CKC response is most noticeable at the central locations of the contralateral hemisphere (C3 and C4), where weaker response components at the stimulation frequency (1.78 Hz) and at its third harmonic (7.12 Hz) are also visible (overall pattern similar as in Piitulainen et al. 2020). In the time–frequency representation the band-like response activation covers the whole epoch duration (two full stimulation cycles) indicating that the response encompasses characteristics common to a steady–state evoked potential. Yet, the parameters chosen for the Morlet wavelet decomposition may also contribute to the appearance.*


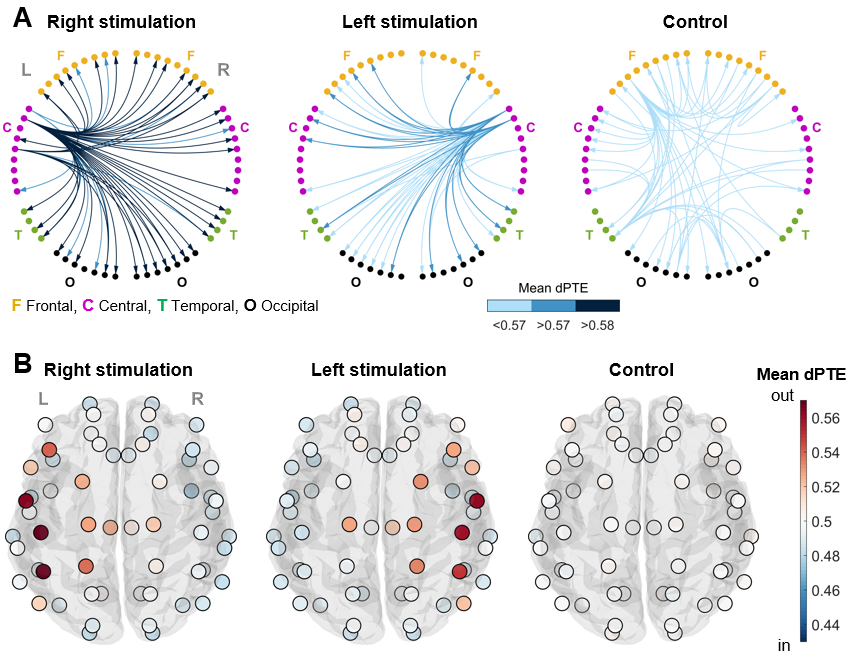


***Figure S5.*** *Supplementary results from the network analysis. Figure A) depicts the strongest functional connections activated by the stimulation when measured with directed phase transfer entropy (dPTE). After averaging dPTEs over the cohort, the strongest 5% of the edges were selected for the circular graph diagrams (conditions separately). The strongest connections activated by the stimulation generate distinct, mutually symmetric patterns similar to the consistent network graphs of Fig. 4B. On the contrary, the connections of the control condition seem highly random. Figure B) shows group averages of the average nodal dPTE values depicted over the 3D brain model. The colour of the circles positioned at center of the parcels represents bias of the nodal information flow (in-/outbound). The data is same as in Fig. 4A with slightly different visualisation.*


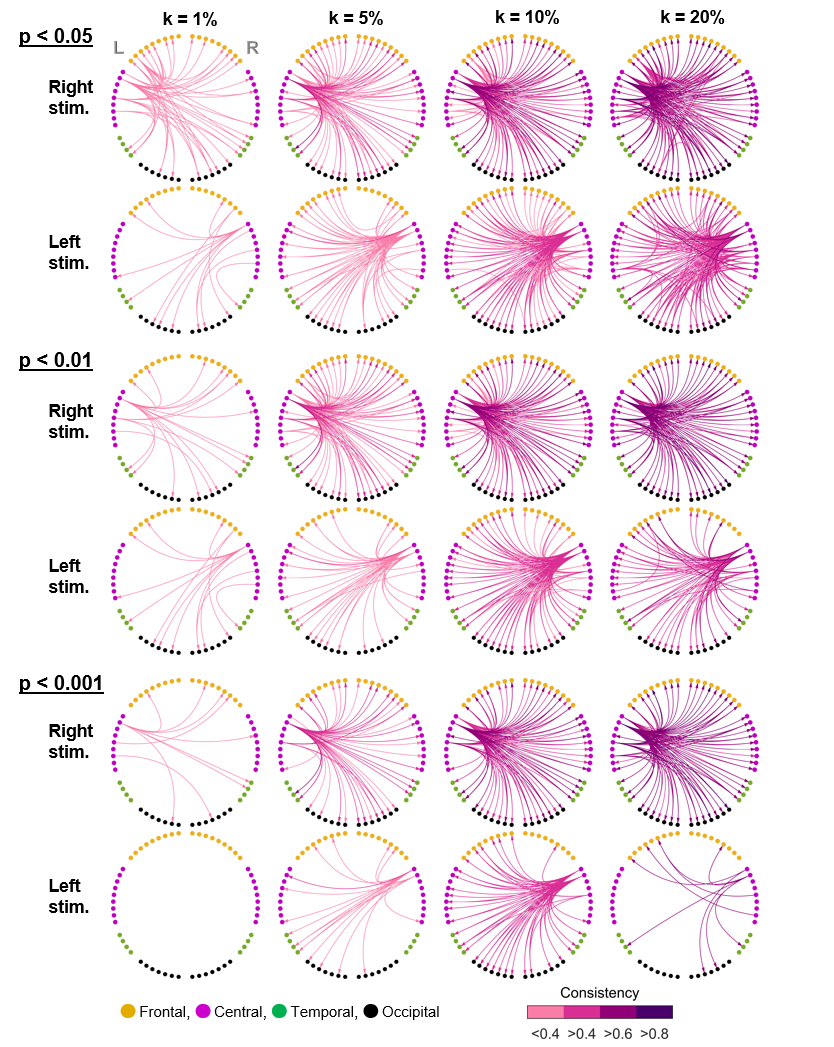


***Figure S6.*** *Consistent Network (CN) graphs with different k-values and p-value thresholds. The circular diagrams show the connections that emerge as the strongest consistently within the recordings during the stimulation. The CN connections are illustrated with a colour scale that depicts the proportion of infants showing the given edge (‘consistency’). Different k-values (1–20%) were used to scale the analysis on specific network sizes, i.e. to constrain the subset of edges that are seen as the strongest. p-value threshold set the alpha level to the subsequent statistical test.*


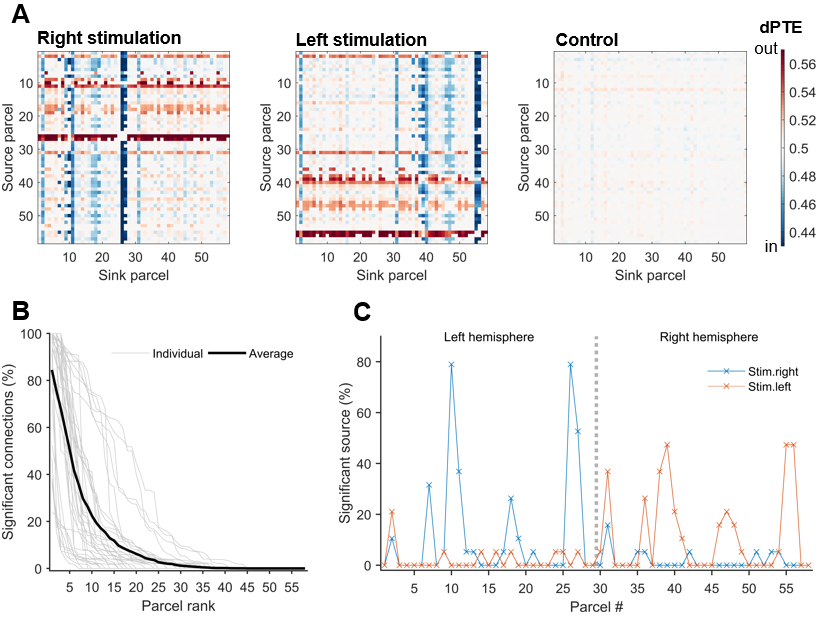


***Figure S7.*** *Significant connections and source parcels. Figure A) shows directed phase transfer entropy (dPTE) values from the three conditions averaged over the cohort and presented as interaction matrices. For the hand stimulations, a few nodes clearly appear as primary information sources generating strongly outbound-biased connections to nearly all other nodes (red toned horizontal lines). We evaluated the significance of the outbound connections at individual level (Spreading Index) with a Z-test using the corresponding surrogate dPTE distribution (control condition) as a reference. Figure B) presents percentages of the significant edges (from all possible edges) for each parcel and recording separately. The parcels (x-axis) are individually organised by their ascending rank, i.e. the one with most significant edges is always positioned on the left. Whereas the strongest parcels recruit on average over 80% of the edges, the effect diminishes steeply, and at the 5^th^ most active parcel, the recruitment is only 50%. Despite the inter-individual variation, the primary source parcels stand out clearly in figure C) that shows how frequently (%) a specific parcel was deemed significant in the cohort. Here we defined a parcel to be significant if it was ranked among the top four, when the proportions of the significant outbound edges were compared. The same limit of four was applied for the calculation of the Spreading Index.*


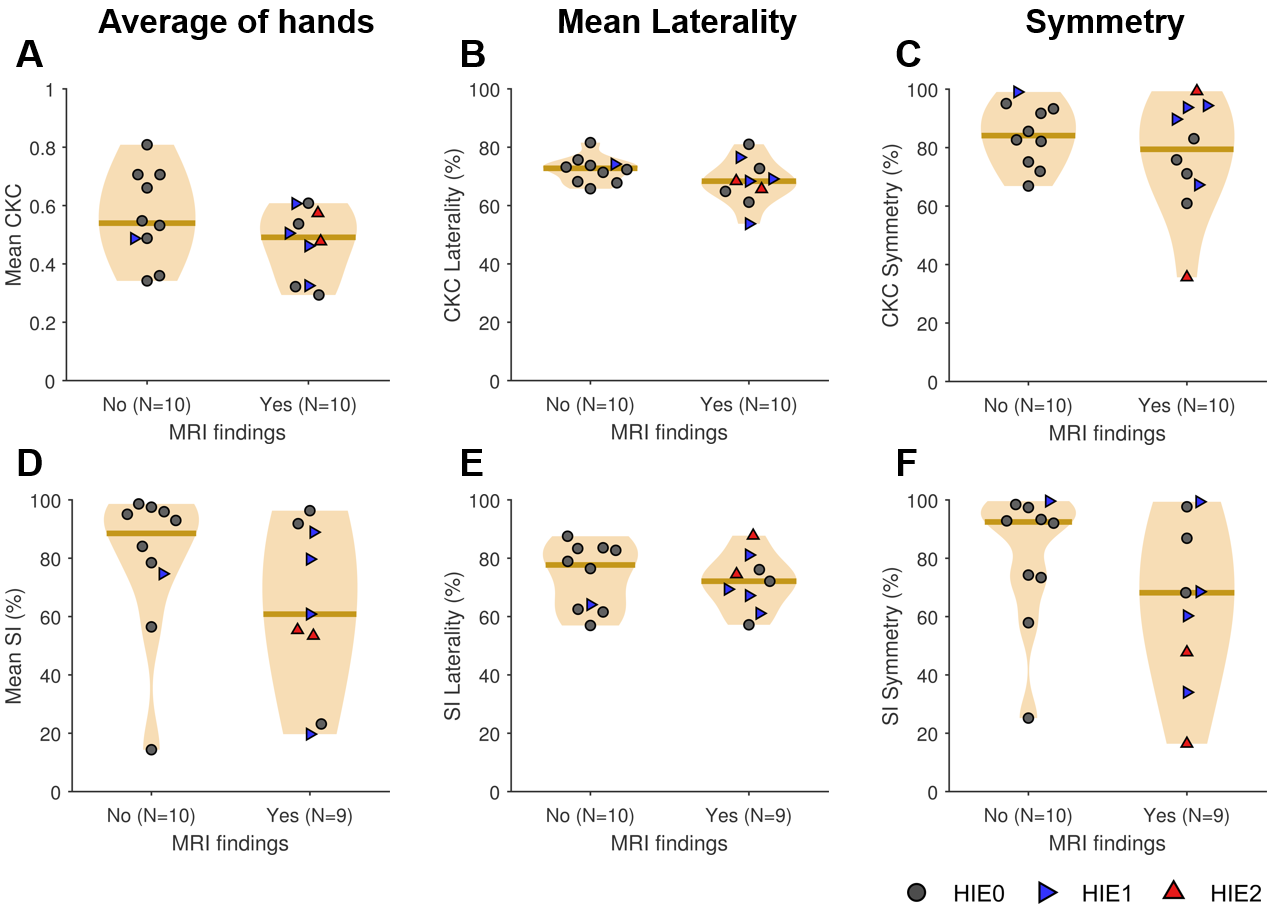


***Figure S8****. Comparison of corticokinematic coherence (CKC) and network metrics of infants based on their magnetic resonance imaging (MRI) results. Figures A-C) depict CKC magnitudes, whereas figures D-F) show Spreading Index (SI) values that quantify the extent of information flow in the networks activated by the stimulation. The infants were divided into two groups based on the evaluation of their MRIs. The horizontal lines represent the median values of these distributions while the different markers of the recordings depict infants in different clinical subgroups (HIE0, HIE1, HIE2). Although, grey and white matter abnormalities were scored separately, here we used a robust classification of whether (or not) MRI contained an abnormal finding of either type. CKC and SI values were assessed based on their average magnitude, mean laterality, and symmetry between right and left side stimulations. For one infant (MRI+), the SI metrics could not be computed due to missing control recording.*

*A recent study (Tuiskula et al. 2022), including partially the same subjects, showed that white matter T2 hyperintensity due to perinatal asphyxia is associated with atypical spontaneous movements in neurological examinations at the age of three months. We hypothesized that there could exist similar relationship also between white matter changes and CKC metrics but could not find statistically significant correlations. Yet, the infants with abnormal findings in their white and/or grey matter showed a slight (non-significant) tendency for lower and more asymmetric SI values compared to those without asphyxia-related abnormal findings in MRI.*

**Pilot recording using a consumer product, electric breast pump, for the CKC stimulation**

In a search for solutions that would allow future scalable CKC implementation in the clinical environments, we wanted to test alternative ways in the stimulation. We carried out a pilot experiment with a commercial breast pump (Philips Avent, Amsterdam, the Netherlands) which is battery-operated and can be programmed to a suitable output frequency. This pump was connected to the balloon used in other parts of this study, and we performed right hand stimulation to a newborn infant at postnatal age of 2 days. The analysis followed our standard signal processing pipeline, and revealed a cortical response consistent with our previous findings within the clinical subgroups and the custom-built stimulator, i.e. the CKC peaked around the contralateral sensorimotor cortex and activated a wide cortical network originating from the same area.


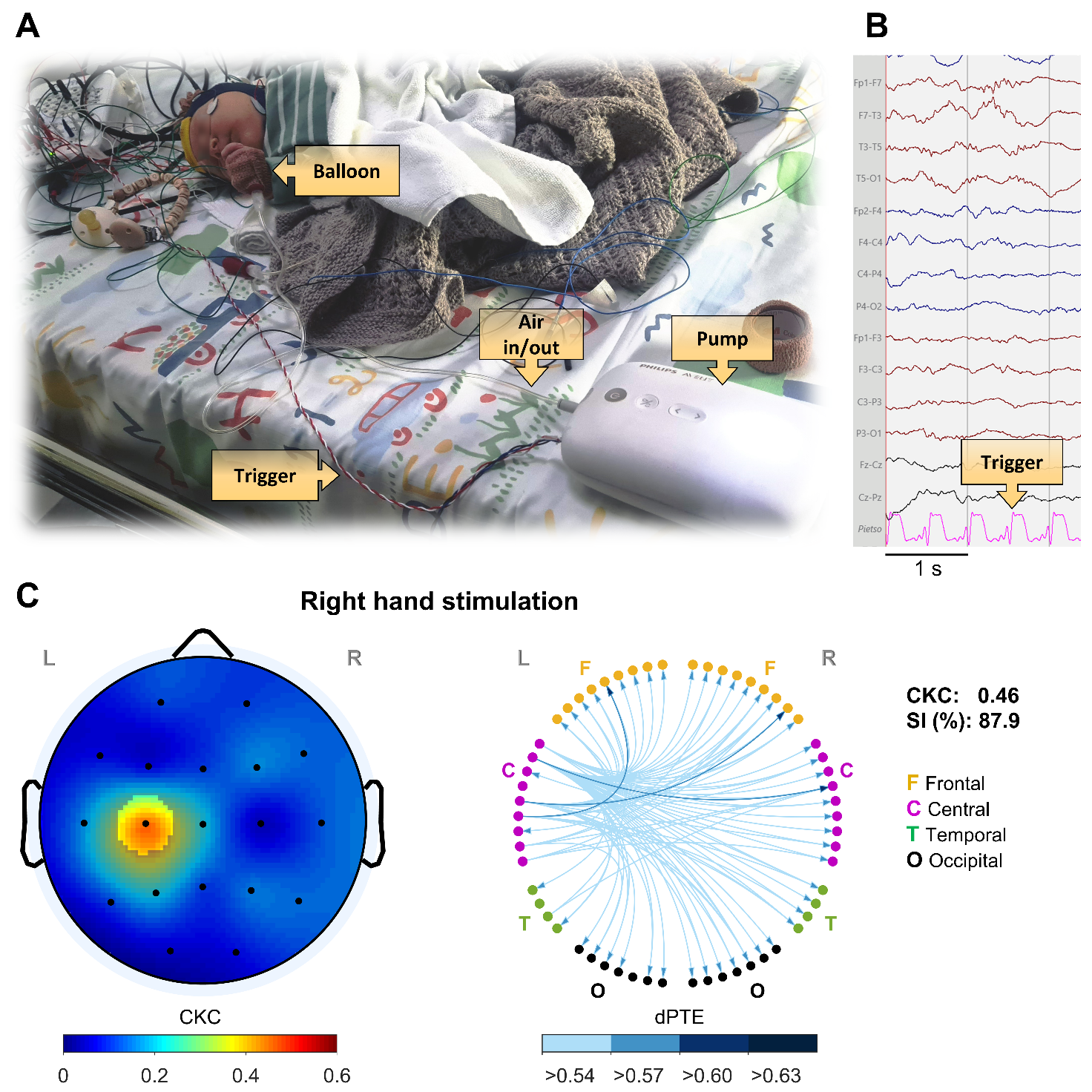


***Figure S9****. A pilot recording using a commercial electric breast pump. Figure A) shows the new stimulation setup where suction of the breast pump generates cyclic hand movements via the balloon wrapped inside the infant’s palm. In the absence of a dedicated trigger output, the synchronisation between the 21-channel EEG acquisition (example in Fig. B) and the stimulation was achieved using a piezoelectric sensor that was physically connected to the pump to record a signal showing the periodic inflation events (see the pink ‘Pietso’ trace in B). The observed interstimulus interval was 500 ms ± 8.1 ms (median ± standard deviation).*

*A right hand movement stimulation (101 epochs, i.e. 111 seconds in total) was performed for a healthy newborn at postnatal age of 2 days (gestational age 41 weeks + 5 days). The signal analysis followed the pipeline described in Fig. 1 and resulted (in Fig. C) in statistically significant (p < 0.01) corticokinematic coherence response peaking at the contralateral C3 electrode location (CKC = 0.46). The related cortical network, calculated using directed phase transfer entropy (dPTE), showed similarly typical recruitment pattern originating mainly from the contralateral central parcels. The associated Spreading Index (SI) yielded a value as high as 87.9%.*


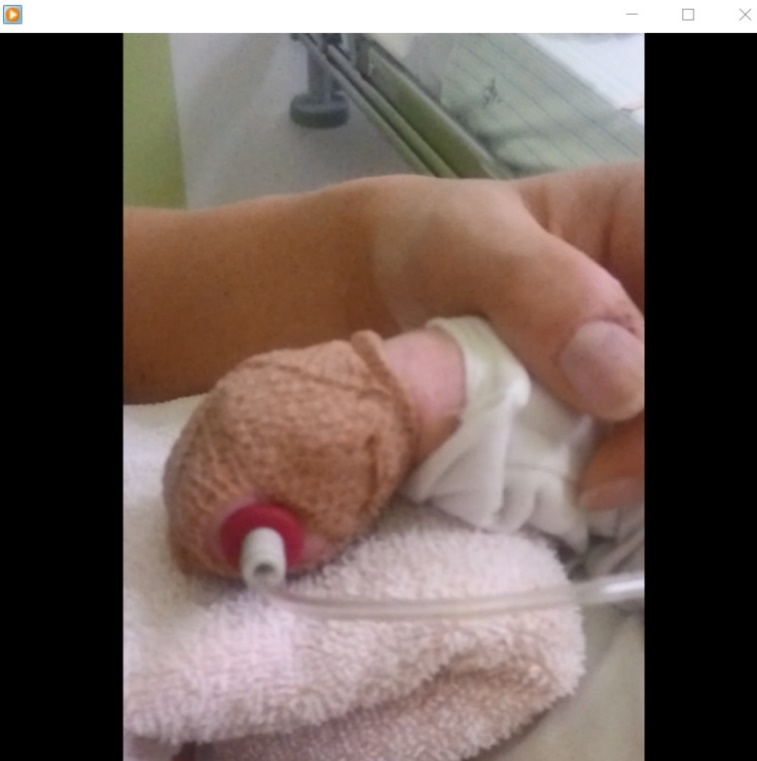


[CKC-hand_stimulation.mp4]

***Video S1.*** *An example of how the pneumatic movement stimulator exerts repetitive passive finger movements via a balloon wrapped to the infant’s palm.*
